# Trade margins of rubber exporters: The case of Indonesia

**DOI:** 10.1371/journal.pone.0292160

**Published:** 2023-11-16

**Authors:** Rossanto Dwi Handoyo, Kabiru Hannafi Ibrahim, Tutus Wahyuni, Fernanda Reza Muhammad, Abdul-Azeez Sani Baraya

**Affiliations:** 1 Department of Economics, Faculty of Economics and Business, Universitas Airlangga, Surabaya, Indonesia; 2 Department of Economics, Faculty of Social and Management Sciences, Federal University Birnin Kebbi, Nigeria; 3 Export Center Surabaya, Surabaya, Indonesia; Universita degli Studi di Foggia, ITALY

## Abstract

This study used a two-step system generalized method of moment (GMM) and spatial aspects to analyze Indonesia’s trade margins of a rubber product to export destination countries over the period 2009–2018. The study unraveled the role of non-tariff measures such as sanitary and phytosanitary (SPS), technical barriers to trade (TBT), and gravity factors in determining rubber trade margins. Our empirical strategies revealed that sanitary and phytosanitary policies negatively affect trade margins, while the technical barrier to trade and foreign direct investment (FDI) asserts a positive impact on trade margins. However, the economics of scale, port, and contiguity increases extensive margin and reduces intensive, population size, distance, and language barrier reduce extensive margin and increase intensive margin. Further evidence revealed that high population size and port quality accompanied by high FDI and distance increases extensive margin and reduces intensive margin. High economics of scale accompanied by distance, port quality, FDI, and population size reduces both trade margins. Our empirical strategy from the spatial analysis does not give overall significant results on each variable as only economies of scale and population size seem to have a spatial influence on trade margins. The study, therefore, recommends that innovation both in terms of technology, like industrial innovation in the field of rubber processing and certification related to rubber commodities, needs to be increased to intensify and expand Indonesia’s rubber market share.

## 1. Introduction

International trade is one form of cooperation between countries to increase national income by way of exports or imports. One factor that underlies the occurrence of international trade is the scarcity of resources. A country is said to be able to export goods or services when the country has a comparative advantage in Wild and Wild [1]. In the international trading system, each country is indirectly required to establish relations with other countries through various agreements and cooperation. The World Trade Organization (WTO) is one of the international trade organizations of which Indonesia is a member country. The purpose of establishing the WTO is to remove barriers to global trade and to promote the flow of export and import Marceau [2]. Reducing tariff barriers has an impact on other restrictions such as quotas and non-tariff measures.

Non-tariff policies, better known as non-tariff measures (NTMs) are policy measures, apart from the customs tariffs, which have the potential impact on international goods trade. Implementing non-tariff policies can change the volume of the goods traded and the prices of the products UNCTAD [3]. There are different kinds of NTMs but in this study, the focus will be on the sanitary and phytosanitary (SPS) and technical barrier to trade (TBT) policies. Broadly speaking, SPS and TBT are different trade policies with different policy targets. The SPS policy is a regulation related to the limitation of goods to be imported by countries to prevent the spread of disease in humans, animals, and plants. This policy covers all the assessment steps for food safety, from certification to testing to quarantine UNCTAD [4].

While the TBT refers to a policy that is more focused on the administrative documents or technical regulations which are not included within the scope of SPS policy. These include; labeling, quality requirements, product characteristics, production techniques, and other measures that protect the environment UNCTAD [4]. These two types of barriers are generally applied to the agricultural sector and animal products. However, TBT can also be implemented in broader industries such as textiles, shoes, processed foods, and chemicals. Henson and Loader [5] in their study show that the requirements of SPS and TBT can hamper trade, especially in the case of developing countries.

Export activity is one of the major indicators of economic growth likewise and commodities traded are also the most crucial component of GDP. An exporting country may be subjected to NTMs by the importing countries in which changes in the value of exports will certainly have an impact on changes in people’s income and living standards. Table 1 shows the trends of Indonesia’s exports. As can be seen from the table, Indonesia’s exports have fluctuated within a few years, and Indonesia is more dominant in exporting non-oil and gas products.

**Table 1:** Indonesia’s exports and imports of oil and non-oil in 2014–2018 (million US$).

Year	Export			Import		
	Oil	Non-oil	Total	Oil	Non-oil	Total
2014	29,975.12	145,748.83	176,036.19	43,416.82	134,585.35	178,002.17
2015	18,571.06	131,768.22	150,366.28	24,592.84	117,984.20	142,577.03
2016	13,105.51	132,028.59	144,489.80	18,722.78	116,809.71	135,532.49
2017	15,744.34	153,083.21	168,827.55	24,313.39	132,653.97	156,967.36
2018	17,191.00	163,024.03	180,215.03	29,863.21	158,812.68	188,675.89

Source: World Bank, World Integrated Solution 2018

The non-oil sector commodity exports, such as the Indonesian plantation sub-sector is affected by the barriers related to NTMs. This is because rubber commodities are closely related to NTMs policies imposed by export destination countries. Indonesia, as an important country in agricultural trade, has the opportunity to expand coverage or increase production. From 2016 to 2018, exports of agricultural commodities, especially the plantation sub-sector, such as rubber, experienced a surge from 2.55 percent to 3.65 percent in 2017. The Ministry of Agriculture of the Republic of Indonesia stated that the plantation subsector provided the highest contribution compared to other subsectors, reaching 387,501.5 billion (in rupiah amount) in 2018. In 2015 rubber production reached 3.15 million tons, which increased to 3.68 million tons in 2017 Ministry of Agriculture Republic of Indonesia [6].

In the world market, demand for Indonesia’s natural rubber commodity amounted to USD 5.1 billion in 2017, which decreased by USD 3.951 billion in 2018 UN Comtrade [7]. Various factors can cause a decline in rubber demand or exports of rubber products. The amount of Indonesia’s rubber exports to the world declined in 2015, and 2016, and increased in 2017. The decline in the value of rubber exports can be caused by competition between major exporters other than Indonesia, such as Thailand as a major exporter of natural rubber and rubber products. In addition to the competition, other things that caused the decline in rubber exports were global rubber prices, which were pressured due to high supply and the decline in demand in China. Rubber prices soared in 2011 and then fell in 2012, 2015, and 2018. The decline is expected to occur due to a rubber surplus. Export prices which were affected by the increase in rubber caused an oversupply so rubber prices weakened. Besides, the weakening demand for products sourced from rubber such as tire demand can also affect world rubber prices. Rubber production mostly comes from small farmers who cannot influence market prices, so when world rubber prices decline, small farmers cannot reduce their production and cause inventories to increase IHS Markit [8].

An increase in the production of rubber products, the amount produced, and the area of rubber plantations have significantly increased the potential of Indonesia to expand the export of rubber products. The development of the amount of rubber production is inseparable from barriers imposed by importing countries such as non-tariff measure barriers to rubber. The main destinations for Indonesia’s rubber exports are mostly the USA, Japan, China, India, Korea, and Germany which imposed heavy NTMs. Considering the role of rubber in Indonesia’s export, high NTMs imposed by export destinations countries, and the lack of clear-cut determinants of rubber export from the available empirical studies. There is a need to examine the determinant of rubber export in Indonesia which is the main focus of this study.

Studies on the impact of NTMs on trade have been widely carried out which include; Bao and Chen [9], Dong and Zhu [10], Fontagné et al. [11], Crivelli and Gröschl [12], Henson and Loader [5], and Hwang and Lim [13], among others. Studies that analyzed the influence of NTMs on extensive and intensive margins have not been conducted to a large extent Ghali et al., [14] and Shepotylo [15]. Felbermayr and Kohler [16] noted that the determinants of the growth and development of intensive and extensive margins are expected to be the formation of new countries (new trading partners) or, in other words, the emergence of the earliest forms of trade between existing countries (old trade policy agreement).

Based on the available empirical studies, the influence of NTMs on trade remained inconclusive as there are many divergent findings. The theory has proposed a negative nexus while available empirical studies provide an inverse or negative nexus and, in some cases, a positive influence prevails. So, there is still ongoing debate about the effect of NTMs on trade which this study is focused to contribute. For instance, a study by Shepotylo [15] states that TBT largely reduces the extensive margin for exports and increases the intensive margin for exports. Another study indicates that NTMs have a more adverse effect on intensive and extensive margins in Egypt compared to other countries Ghali et al. [14]. The differences observed in the findings of many studies exist due to the focus on different commodities, and different trade barrier policies. These are widely discussed among academics and practitioners, and there is a need to explore more about the effect of NTMs especially SPS and TBT on Indonesia’s export margin of natural rubber products.

The choice of rubber in this study is motivated because rubber is major in Indonesia’s plantation products and is mainly export-oriented to many countries around the world. Over the last four decades, Indonesia had experienced substantial growth in rubber production. Rubber plantation is only second to palm oil in generating foreign exchange and is the driver of Indonesia’s non-oil sector. Indonesia is the second leading producer of natural rubber around the world. In Indonesia, there is less government participation in the local rubber industry, and rubber production is dominated by small-scale farmers with poor socio-economic conditions.

In Indonesia, despite the role of rubber products in promoting export and contribution to foreign exchange, there is a lack of empirical studies assessing the determinant of rubber trade margin. Available empirical studies that focus on the effect NTMs mostly focused on the seafood trade Shepotylo [15], tea export Hwang and Lim [13] and Dong and Zhu [10], coffee export Safriani and Priyarsono [17] manufactured and processed food products export Fontagné et al. [11], food and agricultural commodities export Sun and Li [18], Crivelli and Gröschl [12], and Henson and Loader [5], corn export Jayasinghe et. al. [19]. Other studies focus on the aggregate trade flow Bao and Chen [9] and Bao and Qiu [20]. The only existing study that focused on rubber export includes; Virginia [21]. Therefore, there is a need to assess the influence of NTMs on the trade margin for rubber export in Indonesia. This is because, despite the expanding trade in rubber and being among the top exporters of rubber products, the determinants of Indonesia’s rubber export are still not known.

Therefore, the contribution of this study lies in an examination of the effect of NTMs particularly SPS and TBT on the extensive and intensive margins by incorporating spatial aspects (i.e. Geographic Information System) using the dynamic panel. The study will also analyze the interaction variables between demand factors (economies of scale and population variables) and other variables to see how strong the influence of demand variables is on other variables. It is interesting to study that rubber commodities are commodities based on natural resources and are very vulnerable to changes in demand for these commodities. It has been noted that export products based on natural resources (primary products) have an elastic characteristic of export demand and an inelastic export supply Carbaugh [22].

This study is structured into five sections. Section one explains the background of the problem which includes the main issue as well as the purpose of the study. The second section explains the available literature to serve as a basis for answering the research problems, and the method as links to topics and variables used in the study. Section three discusses the research method. Section four presents the empirical finding. Lastly, section five presents the conclusion and offered policy recommendations.

## 2. Review of literature

### 2.1. Extensive and intensive margin

Dutt et al. [23] explain the method of breaking down extensive margins to calculate variations in the number of export products and new export companies, whereas intensive margins depend on the average trade value per product category or value per export company. This method as used by Dutt et al. [23] has some shortcomings while in this study we used a technique of measuring trade margin as proposed by Hummels and Klenow [24] popularly known as the HK method. This method has the advantage of correcting the bias associated with the dominance of one single product or several products traded between countries. Based on the work of Said and Ismail [25] and Hummels and Klenow [24], the method of measuring extensive margins is as follows:

$$EM_{ijt}^{m}=\frac{\sum m\in M_{ijt}\sum X_{jwt}^{m}}{\sum m\in M_{wjt}X_{jwt}^{m}}$$ (1)

Where: *EM*^*m*^_*ijt*_ is an extensive margin calculated based on the import value of the product of country _*j*_ from _*i*_ in year *t*. *X*^*m*^_*jwt*_ is the value of the import of country _*j*_ products from all countries in year _*t*_. *M*_*ijt*_ is a subset of all products imported by country _*j*_ from country _*i*_ in year _*t*_. *M*_*iwt*_ is a collection of all products imported by _*j*_ from all countries in the year _*t*_. The calculation of the intensive margin of a product imported by country _*j*_ from country _*i*_ is:

$$IM_{ijt}^{m}=\frac{\sum m\in M_{ijt}\sum X_{jwt}^{m}}{\sum m\in M_{wjt}X_{jwt}^{m}}$$ (2)

Where: *IM*^*m*^_*ij*_ is the intensive margin and all other variables were as explained in Eq (1).

The classification of NTMs is divided into two and broken down into several chapters depending on the scope and technical measures such as SPS, TBT, pre-shipment inspection, and other non-technical measures. A policy in the form of sanitary and phytosanitary (SPS) is a policy or action used to protect imported or exported goods and services from the risk of being exposed to additives, poisons, or disease-causing organisms. Technical trade barriers (TBT) are regulations that aim to ensure that technical regulations and packaging standards, labeling, and conformity assessment procedures are not discriminatory and do not cause other obstacles in international trade. This TBT policy can not only be applied to agricultural products but can also be used for more general products UNCTAD [3].

### 2.2. Gravity model

The gravity model in international trade is a model used to analyze the flow patterns and volumes of trade in goods or services between two or more countries by combining geographical distances in the model Susetyo and Handoyo [26]. Tinbergen [27] and Pöyhönen [28] first developed the gravity model, which was adapted based on the analogy of Newton’s universal law of gravity. Tinbergen’s [27] research results show that international trade will be proportional to the economic capacity of each country because the occurrence of international trade is mainly expressed in the gross domestic product (GDP). In the gravity model, other than the GDP of the importer and exporter as a trade factor, the distance between the exporter and the importer is a proxy of trade costs.

### 2.3. Aspects of spatial interaction

The influence of neighborhood in a formulated empirical model is addressed using a spatial econometric model. In international trade, geographical proximity will result in high trade intensity between countries. This, therefore, implies that countries that are located in the same region will tend to have higher trade intensity relative to countries that are geographically far apart. The longer the distance between countries the higher the transport costs and this negatively affects trade. So, it is important to include spatial aspects in the context of this study. According to Anselin [29] estimating spatial impact can be made in various ways, one of which is using Moran’s index, which then sees the level of significance. The Moran index equation is as follows:

$$I=\left[\frac{n}{\sum_{i,j}W_{ij}}\right]\frac{\sum_{i,j}W_{i,j}\left(x_{i}-\bar{x}\right)\left(x_{j}-\bar{x}\right)}{\sum_{i}\left(x_{i}-\bar{x}\right)}$$ (3)

Where: $\bar{x}=\left[\frac{1}{n}\right]\sum_{i}\;x_{i}$ and *W*_*i*,*j*_ are weighting techniques. In a diagonal weighting matrix (*w*), the main diagonal will be *0* because the area does not have neighbors or is only next door to itself. Then other cells that can be valued more than *0* identify the area that has contiguity with other regions. The higher the weighting value, the stronger the effect compared to the observed area Pohan [30].

The spatial aspect of international trade can be traced back to the theory which states that countries that are geographically close to each other would tends to have high trade intensity. This implies that countries that are within the same region will tend to have higher trade relations than countries that are geographically far apart. The longer the distance between countries the higher the transport costs and this negatively affects trade. So, it is imperative to consider the spatial aspect in this study because of the spatial variation and spatial independence. Due to the geographical proximity among our sample countries, there is a tendency for the existence of spatial autocorrelation. Hence, the presence of spatial autocorrelation will violate the basic assumption of residual independence and render the statistical estimate invalid. Therefore, there is a need to consider spatial econometrics because with the spatial variation conventional econometric techniques become unsuitable for the estimate.

### 2.4. The models

Following Shepotylo [15] and Sun and Li [18], in this study, we used a modified gravity model. The use of the gravity model in this study is necessitated by the fact that since the ground-breaking work of Melitz [31], empirical studies had extensively used the gravity model to explain the patterns and margins of trade. Therefore, the trade margin (extensive and intensive) of Indonesia’s rubber export is expressed in the following models

$$\;TMargin_{ijt}=\alpha +\beta_{1}\;CRTBT_{ijt}+\beta_{2}\;CRSPS_{ijt}+\beta_{3}lnECS_{ijt}+\beta_{4}FDI_{ijt}+\beta_{5}X_{ijt}+\mu_{i}+{ʎ}_{t}+\varepsilon_{ijt}$$ (4)

Where: *TMargin*_*ijt*_ is the trade margin which includes the extensive margin (*EM*) and the intensive margin (*IM*), *α* is the model intercept, *CRTBT* is the coverage ratio of technical trade barriers (*TBT*),*CRSPS* is the coverage ratio of the sanitary and phytosanitary (*SPS*).*ECS* is the economies of scale in log form. *ECS* is measured by the ratio of the GDP of destination countries to Indonesia’s GDP. The *ECS* reflects the ability of countries to produce goods and buy goods from other countries and this can positively affect both the extensive and intensive margins. *FDI* is the foreign direct investment, *X*_*ijt*_ is the vector of geographic factors. These geographic factors include variables such as; distance *DIST*_*ijt*_ in log form. Distance may reflect the transport costs as the theory posits. The higher the distance, the higher the transport costs and the lower the extensive and intensive margins. common language dummy *LAN*_*ijt*_, the quality of the seaport index *PORT*_*ijt*_.*POP*_*ijt*_ is the population size which is the ratio of the total population of destination countries to exporting countries (Indonesia). *CONTIG*_*ijt*_ is the dummy of the contiguity variable. This variable is important if the destination country is adjacent to Indonesia and is expected to increase both the extensive and intensive margins. The *β*_*s*_ i.e. *β*_1_ …… *β*_5_ are the parameters to be estimated, _*j*_ is the importer country, _*i*_ is the exporter country, μ is the unobserved effect that is specific to individual unit or export, ʎ_*t*_ is the time effect common to all the exporters, and *ε* is the conventional idiosyncratic error term that is assumed to be independently and identically distributed.

Provided that the cross-country effect (μ_*ij*_) is constant and there is a non-appearance time effect (ʎ_*t*_ = 0), the Model (4) can be estimated using restricted ordinary least squares (OLS). With random disturbance term, constant country effect, absence of time effect, and with zero expectation of error term such that *E*(œ_*i*_) = 0, with variance (œ_*i*_) = σ_œ_^2^ and covariance (ε_*i*_,œ_*i*_) = 0. With these assumptions Eq (4) can simply be estimated using Generalized Least Squares (GLS) or Random Effect (RE) models. However, with the assumption that the country effect is constant and equal across sample countries i.e. μ = ʎ_*t*_ and ʎ_*t*_ ≠ 0, the Model (4) can be estimated using the Fixed Effect (FE) model.

As the associations between most economic variables are dynamic and the possible problem of endogeneity which the POLS, RE, and FE models cannot solve, we saw the need to apply the dynamic model with the Generalized Method of Moment (GMM) estimator as proposed by the Arellano and Bond [32]. In a dynamic modeling approach, the trade margin of rubber exporters and its determining factors which varies across space and time can be expressed as in Model (5):

$$\;TMargin{\;}_{ijt}=\alpha +\beta_{0}\;TMargin_{ij(t-1)}+\beta_{1}\;CRTBT_{ijt}+\beta_{2}\;CRSPS{\;}_{ijt}+\beta_{3}lnECS_{ijt}+\beta_{4}FDI_{ijt}+\beta_{5}X_{ijt}+\mu_{i}+{ʎ}_{t}+\varepsilon_{ijt}$$ (5)

Where:*α*, *TMargin*_*ijt*_, *CRTBT*, *CRSPS*, *ECS*, *FDI*, *X*_*ijt*_, *DIST*_*ijt*_, *LAN*_*ijt*_, *PORT*_*ijt*_, *POP*_*ijt*_, *CONTIG*_*ijt*_, μ_*i*_, *ʎ*_*t*_, *ε*_*ijt*_, and *β*_*s*_ are as defined in Eq (4). *TMargin*_*ij*(*t*-1)_ is the lagged dependent variable incorporated to capture dynamic aspects of the estimated model. To remove the country-specific effect in Eq (5) Arellano and Bond’s [32] approach required taking the first difference of Eq (5) as follows:

$$TMargin_{ijt}\;=\;\alpha \;+\;\beta_{0}\Delta TMargin_{ij(t-1)}\;+\;\beta_{1}\Delta CRTBT_{ijt}\;+\;\beta_{2}\Delta CRSPS_{ijt}+\beta_{3}ln\Delta ECS_{ijt}\;+\;\beta_{4}\Delta FDI_{ijt}\;+\;\beta_{5}\Delta X_{ijt}\;+\;\Delta {ʎ}_{t}\;+\;\Delta \varepsilon_{ijt}$$ (6)

The variables in Eq (5) are as defined in Eqs (4) and (5) except that they entered into Eq (6) as the first difference. The country-specific effect (μ_*i*_) has been eliminated by differencing in Eq (6). In this equation, the within estimator is said to be biased while differencing the remove the country-specific effect. This is because of the differences in the lagged dependent variable which is assumed to be correlated with the differences in the random error term. Due to this problem, when the series is persistent and with a small period the difference GMM estimate would be biased due to weak instrumentation from the lagged levels. When the difference GMM estimate is biased due to weak instrumentation resulting from a persistent lagged dependent variable, the most preferred estimation method is the system GMM estimate as proposed by Blundell and Bond [33]. If the data persistency is weak, the difference GMM estimator is likely to be less biased and more appropriate because it would possess some efficiency gain than the system GMM estimate.

As stated previously, our formulated model would be estimated using the dynamic GMM estimator since by implication the model is a complicated equation due to the link between the lagged variable (*TMargin*_*ij*(*t*-1)_) and the idiosyncratic error term (*ε*_*ijt*_). This issue is automatically resolved in GMM estimated using moment conditions. A valid estimate requires that the expected value of the dependent and independent variables in a given period is independent of the error term. That is the covariance of the error term and any of the independent variables is zero. To sum it all up, the dynamic panel data modeling approach is based on the assumption that;

- Across time and individual countries, the error terms (*ε*_*ijt*_) are independently and identically distributed (i.i.d.) with zero mean, that is *E*(*ε*_*ijt)*_) = 0 and $Var\left(\varepsilon_{ijt}\right)=\sigma_{\varepsilon }^{2}$.
- Across time the ʎ_*t*_ are i. i. d. with *E*(_ʎ*t*_) = 0 and *Var*(ʎ_*t*_) = σ_ʎ_^2^.
- The μ*i* in Model (5) is also assumed to be i. i. d. across individual countries with *E*(μ_*i*_) = 0 and *Var*(μ*i*) = σ^2^_μ_.
- The error terms (ε_*ijt*_) component are assumed to be orthogonal to exogenous variables, that is; *E(x′*_*it*_ *ε*_*it*_*)* = 0.
- The individual effect (μ_*i*_) might be correlated with the exogenous variables in the model, that is; *E(x*_*it*_*′u*_*i*_*) ≠ 0*.
- The endogenous lagged independent variable is uncorrelated with the error terms, that is; *E*(*TMargin*_*ij*(*t*−1)_, ε*ijt*) = 0.

Additionally, the parameters of the model were estimated using a two-step system GMM. This is important in a dynamic model because theoretically, the two-step estimate has the best balancing matrices that are more robust and efficient than one step. That is, the GMM applied the weighting matrix for the one-step estimate and used its residuals as the weighting matrix for the two-step estimator Youssef et al. [34]. In addition to our baseline gravity model, different models were also estimated that include interaction terms of different economic and geographic factors. This is important in ensuring the robustness of the empirical findings and checking for the stability of estimated parameters. We derived from the baseline model, different models that include (*POP* × *FDI*) interactions, (*POP* × *PORT*) interactions, (*POP* × *DIST*) interactions, (*PORT* × *FDI*) interactions, (*ECS* × *DIST*) interactions, (*ECS* × *PORT*) interactions, (*ECS* × *FDI*) interactions, and (*ECS* × *POP*) interactions. Table 2, therefore, shows different models as derived from the baseline model. These models are indicated in columns 2–9 and are estimated by including a different set of interaction variables.

**Table 2:** Extensive and intensive margins models with different interaction variables.

Variables/Model	1	2	3	4	5	6	7	8	9
*CRTBT*	✓	✓	✓	✓	✓	✓	✓	✓	✓
*CRSPS*	✓	✓	✓	✓	✓	✓	✓	✓	✓
*ECS*	✓	✓	✓	✓	✓	✓	✓	✓	✓
*FDI*	✓	✓	✓	✓	✓	✓	✓	✓	✓
*DIST*	✓	✓	✓	✓	✓	✓	✓	✓	✓
*LAN*	✓	✓	✓	✓	✓	✓	✓	✓	✓
*PORT*	✓	✓	✓	✓	✓	✓	✓	✓	✓
*POP*	✓	✓	✓	✓	✓	✓	✓	✓	✓
*CONTIG*	✓	✓	✓	✓	✓	✓	✓	✓	✓
*POP × FDI*		✓							
*POP × PORT*			✓						
*POP × DIST*				✓					
*PORT × FDI*					✓				
*ECS × DIST*						✓			
*ECS × PORT*							✓		
*ECS × FDI*								✓	
*ECS × POP*									✓

Note: Indicates that the marked variable is included in the corresponding model as described in model 4 and presented in Tables 4 and 5.

### 2.5. Empirical review

Existing theories posit a negative link between SPS, TBT, and extensive and intensive trade margins. SPS and TBT policies negatively affect the productivity of exporting countries. This effect will result in an increase in production due to standardization and other procedures that must be met to export to the destination countries. A study by Virginia [21] reports that SPS negatively affects Indonesia’s rubber export while TBT positively affects rubber export to destination countries. Another study by Shepotylo [15] observed that SPS positively affects the extensive margins and negatively affects the intensive margins of seafood world export. Furthermore, findings indicate that TBT negatively affects extensive margins and positively influences intensive margins. In a sample of 103 and 105 countries, Bao and Chen [9] and Bao and Qiu [20] have found TBT to significantly increase extensive margin. Over the period 2005–2011 Kamal and Zaki [52] report no significant effect of TBT on Egyptian firms’ intensive margin while extensive margin is negatively affected by TBT. However, their finding indicates that smaller firms are more affected by the negative effect of TBT. Ghali et al. [14] have found that NTMs have a more robust impact in Egypt while there is no insignificant effect in Tunisia. Over the period 1992 to 2013 Dong and Zhu [10] used gravity and examined the effect of the SPS gap between China and its tea export destination countries. Their findings revealed that China’s tea export is restricted by a high gap in SPS measures from its ten developed tea trading partners. Safriani and Priyarsono [17] also found a negative effect of SPS and TBT on Indonesia’s coffee export. Susetyo and Handoyo [26] used the gravity model and examined the effect of trade facilitation on extensive and intensive margins in the 8 ASEAN-China free trade areas (ACFTA) for the period 2006–2014. Their finding indicates a significant positive effect on both extensive and intensive margins.

Dutt et al. [23] investigate the effect of GATT and WTO membership on trade margin. Their empirical strategy based on gravity revealed a significant effect on extensive and intensive margins. Similarly, Felbermayr and Kohler [16] support WTO membership effect on the extensive and intensive margins. Fontagné et al. [11] observed an insignificant positive effect of SPS and TBT on manufactured goods and a significant negative effect on fresh and processed food products. Crivelli and Gröschl [12] have found the tendency for SPS to reduce trade in food and agricultural commodities. Contrarily, in a sample of developed and developing countries Henson and Loader [5] report that the SPS has enabled developing countries to realize their potential for food and agricultural commodity exports to developed countries. Hwang and Lim [13] examined the effect of differences in SPS based on the stipulated maximum residue levels (MRLs) and report that variations in maximum residue levels (MRLs) have negatively affected tea exports between countries. Jayasinghe et. al. [19] have found that US corn export to 48 sample countries is negatively affected by SPS measures over the period 1989–2004. For the period 2000–2015, Sun and Li [18] examined the determinants of Chinese agricultural export margins to the ASEAN countries. Their findings revealed that the Chinese extensive margin has moved to an intensive margin after the establishment of the China–ASEAN Free Trade Area CAFTA.

### 2.6. Hypothesis development

Based on previous studies and theoretical underpinnings many hypotheses were formulated to verify the relationship between the variables. These hypotheses include:

- Allegedly non-tariff measures (SPS and TBT) imposed by importing countries on rubber commodities have a significant negative effect on extensive and intensive margins.
- It is suspected that the economies of scale and population size of countries that trade rubber have a positive and significant effect on extensive and intensive margins.
- It is suspected that the distance between the countries that trade rubber has a negative and significant effect on the extensive and intensive margins.
- It is suspected that the language of the countries that trade rubber has a positive and significant effect on extensive and intensive margins.
- It is suspected that port quality has a positive and significant influence on extensive and intensive margins of rubber.
- It is suspected that the interaction variable can increase the export margin of Indonesia’s rubber trade.
- There is spatial interaction in the rubber trade carried out by the destination countries. This means that the distance closer to other countries will have a stronger influence on trade compared to countries far away. So that it has an impact on increasing extensive and intensive margins.

## 3. Data and estimation techniques

This study used a quantitative approach to analyze the effect of non-tariff measures on extensive and intensive margins over the period 2009 and 2018. The study also used a sample of 23 countries that mostly import Indonesia’s rubber and analyze the spatial aspects and interactions of other explanatory variables. The countries include; Cambodia, Malaysia, Philippines, Singapore, Thailand, Vietnam, Australia, China, India, Japan, New Zealand, Korea, USA, Turkey, Brazil, Germany, France, Netherlands, Spain, Belgium, United Kingdom, and Italy. Stata and GeoDa were used for the analysis.

The operational definition of each variable used is as follows.

- Extensive and intensive margins are the respective export growths derived from an increase in new products or more varied products as well as an increase or decrease in the number of products exported using the Hummels and Klenow [24] method. The method produces the value of the margins in the form of a ratio that ranges from *0* to *1*.
- SPS barriers in this study are used as dummy variables and are calculated using the following formula;

$$C_{ijt}\;=\;\left[\frac{\sum_{k=1}^{n}\left(D_{kt}V_{kt}\right)}{\sum_{k=1}^{n}V_{kt}}\right]$$ (7)

Where: *C*_*ijt*_ is the coverage ratio of country _*i*_ to country _*j*_ on _*t*_ (in percentage), *D*_*kt*_ is the dummy variable (with a value of *1* if there are NTMs in product _*k*_ in year _*t*_ and *0* otherwise), *V*_*kt*_ is the value of product _*k*_ imports in year *t*.

- TBT is a standard or technical procedure for assessing whether the product complies with existing processes. Similar to the SPS policy, the TBT variable is also calculated using a coverage ratio. The coverage ratio has a value between *1* to *100*.
- Economies of scale are calculated as the ratio of the GDP per capita of the importing country to the GDP per capita of the exporting country. *ECS* indicates the ability of the population to produce and consume the products. A percentage rise in *ECS* could result in a certain percentage rise in export.

$$ECS=GDP_{j}/GDP_{i}$$ (8)

- The population is measured by the total population of the destination country as against Indonesia’s population. This is used to reflect the level of market potential as well as the potential of ur to produce goods and services.
- Language similarity is a dummy variable used to categorize the taste of the importing country toward goods from the exporting country.
- Geographic distance illustrates the trade cost of the exporting country and the importing country. The data for this variable is transformed into natural logarithms.
- Port has measured the quality of port infrastructure in international trade. The port quality is an index value that ranges from *0* to *7*. A better and quality port infrastructure that supports a trading system will have an index closer to 7.
- Contiguity is a dummy variable that indicates whether an importing country is directly adjacent to Indonesia or otherwise.
- Foreign direct investment is measured as a percentage of *GDP*.
- The interaction terms between *ECS* and *POP* variables, *ECS* with *FDI*, *ECS* with *PORT*, *POP* with *FDI*, *POP* with *PORT*, *PORT* with *FDI*, *ECS* with *DIST*, and *PORT* with *DIST*. These variables are the results obtained by multiplying a variable with a given variable. This is important in measuring the indirect effect of different variables, and the robustness of the empirical findings.

### 3.1. Data source

As mentioned earlier, the study used panel data consisting of 23 countries over the period 2009–2018. The data for this study were sourced from sources that include; the market access map of the International Trade Centre (ITC), World Bank, World Development Indicator, World Bank, World Integrated Trade Solution, and the Centre d’etudes prospectives et d’informations internationales (CEPII). The data for coverage ratio SPS (CRSPS) and TBT (CRTBT) were sourced from the market access map of the International Trade Centre (ITC). The data for economies of scale, population, port, and FDI are sourced from the World Bank, World Development Indicators (WDI) database. The data for language, geographical distance, and contiguity are sourced from the Centre d’etudes Prospectives et D’informations Internationales (CEPII).

### 3.2. Dynamic panel

The study used dynamic and spatial panel data regression with GMM and spatial error models (SEM) methods in geodata. The GMM is used for the dynamic panel data. The use of panel data in this study is necessary and important in overcoming the problems of data shortages and endogeneity. In using the GMM method, there are two estimates of the autoregressive linear model which are the first difference and system GMM estimates. Although, some studies by Youssef and Abonazel [35], Youssef et al. [34], Hayakawa [36], and Han and Phillips [37] have attempted to improve the efficiency of GMM. We choose to use the system GMM estimators instead of difference GMM because with weak instruments and large moment conditions the first difference GMM can suffer from considerable sample bias Abonazel and Shalaby [38].

The GMM estimate must meet the requirements of specifications tests which are used to validate the consistency of model variables. These tests are the Sargan and Hansen tests and the Arellano and Bond [32] AR test. The system GMM is an extended version of the first difference in which the equation in the first difference is combined with the level equation so that the variables used remain orthogonal to *λ*_*i*_. Blundell and Bond [33] stated that in the autoregressive distributed lag model, the series from the first differenced could not correlate with records having average stationarity. The system GMM estimator combines a set of standard equations in a first difference form with a level form dependent variable that is suitable as an instrument. Although the *Y*_*it*_ level is correlated with *μ*_*it*_. The system GMM requires that first differences are uncorrelated, thus allowing lagged first differences to be used as an instrument for equations in the levels form. As stated earlier, we used the two-step system GMM instead of the one-step because the two-step uses the best balancing matrices that are more robust and more efficient than the one-step. Again, many recent studies like Ibrahim et al. [39], Ibrahim et al. [40], Ibrahim [41], Ibrahim et al. [42], and Handoyo et al. (2022) [43] have also applied two-step GMM for analysis of the dynamic phenomenon.

### 3.3. Spatial panel

Following recent studies by Youssef et al. [44] and Youssef et al. [45], we used spatial data with the effect that considers aspects of space/fields. Spatial data is more closely defined as data with locations that have close distances compared to information that has longer distances Cressie [46]. Spatial data is divided into three types, such as (1) point-referenced data, (2) area data, and (3) point-pattern data. Point-referenced data is usually called geostatistical data. According to Anselin [29] to conduct spatial analyses, the initial step is to determine the weighting matrix (*w*) or see the coordinates of the location (longitude, latitude). There are several methods for defining the relationship between the distance or neighborhood contiguity which include among others; rook contiguity, bishop contiguity, and queen contiguity.

After determining the weighting (*w*) or the coordinates of the location, the Moran I test is used to see whether there is a spatial influence in the model. The spatial dependency test hypothesizes that if the probability value is smaller than its level, then there is a spatial effect on the model. Spatial models are divided into general spatial models (GSM), spatial autocorrelation models (SAR), spatial error models (SEM), general nesting spatial models (GNM), spatial autocorrelation (SAC), spatial Durbin models (SDM), spatial Durbin error models (SDEM), spatial lag of X (SLX) Elhorst and Vega [47]. The econometrics model for the spatial regression model above can be written using the following equation Elhorst and Vega [47].

### 3.4. Spatial autocorrelation model (SAR)

Models that include spatial variables and responses can be expressed in the following form;

$$y_{it}\;=\;\alpha +\beta_{1}\sum_{j=1}^{N}w_{ij}\varepsilon_{it}+\beta_{2}+\varepsilon_{it}$$ (9)

### 3.5. Spatial error model (SEM)

Models that include spatial variables in error terms can be expressed in the following form;

$$y_{it}=\alpha +\beta_{1}+\beta_{2}+\varepsilon_{it}$$ (10)

In Models (9) and (10) which are the spatial autocorrelation (SAR) model and the spatial error model (SEM) the distribution of the error term is such that, there exists autocorrelation across the observed data. In the models, it is assumed that there is spatial dependence in the error term (ε_*it*_) which is of the spatial unit and corresponds to adjacent units. A model with this distribution is called the spatial autoregressive (SAR) model with spatial autoregressive error (SAR). This kind of spatial dependence cause the estimate of the model parameters to be biased and inconsistent using the ordinary least square (OLS) model. The SAR model can be obtained if the autoregressive coefficient in Model (9) is non-zero and the spatial autoregressive is zero.

## 4. Empirical results and discussion

As stated earlier, using 23 export destination countries, the purpose of this study is to examine the determinants of Indonesia’s rubber export trade margin (i.e. both the extensive and intensive margin). Additionally, the study also examines the spatial and interactive factors that influence the dependent variables (i.e. extensive and intensive margins). The empirical strategies are reported in Tables 3–6.

**Table 3:** Descriptive statistics.

Variable notation	Observation	Mean	Min	Max	Std dev.
*EM*	230	0.018	0.001	0.065	0.017
*IM*	230	2.638	0	189.91	17.632
*ECS*	230	3.13e+08	4.222	1.94e+09	4.58e+08
*POP*	230	0.711	0.176	5.579	1.424
*DIST*	230	8005.2	886.141	16371.12	4832.943
*FDI*	230	4.380	-26.195	41.919	7.532
*CRSPS*	230	0.413	0	24.378	0.413
*CRTBT*	230	0.648	0	40.231	3.861
*PORT*	230	4.503	0	6.8	1.815
*CONTIG*	230	0.043	0	1	0.204
*LANG*	230	0.087	0	1	0.282

Source: Authors’ calculation

**Table 4:** Correlation matrix and variance inflation factor (VIF).

	EM	IM	CRTBT	CRSPS	ECS	FDI	DIST	LANG	PORT	POP	CONTIG
EM	1										
IM	0.05	1									
CRTBT	-0.11	-0.07	1								
CRSPS	-0.07	-0.07	0.78	1							
ECS	-0.36	-0.39	0.13	0.07	1						
FDI	0.15	-0.02	0.01	0.04	-0.32	1					
DIST	-0.84	-0.08	0.08	0.04	0.46	-0.27	1				
LANG	0.50	-0.03	-0.08	-0.04	-0.21	0.32	-0.67	1			
PORT	-0.07	-0.02	0.03	0.05	0.00	0.27	0.05	0.17	1		
POP	0.01	-0.10	0.14	0.05	0.58	-0.14	-0.00	-0.14	-0.14	1	
CONTIG	0.41	-0.05	-0.06	-0.05	-0.05	-0.03	-0.43	0.69	0.06	-0.09	1
VIF			2.74	2.68	2.68	1.51	3.13	3.96	1.18	2.12	2.24

Source: Authors’ calculation

**Table 5:** Two-step GMM estimated results of extensive margins of rubber export.

Variables	Model 1 Two-step GMM	Model 2 Two-step GMM	Model 3 Two-step GMM	Model 4 Two-step GMM	Model 5 Two-step GMM	Model 6 Two-step GMM	Model 7 Two-step GMM	Model 8 Two-step GMM	Model 9 Two-step GMM
*L*.*EM*	0.821***	0.854***	0.835***	0.861***	0.876***	0.845***	0.837***	0.847***	0.756***
*SPS*	-0.0001***	-0.000***	-8.35e-05**	-0.000***	-8.34e-05**	-8.49e-05**	-0.000***	0.000***	-0.000***
*TBT*	0.0001***	0.000***	0.000***	0.000***	9.36e-1***	9.13e-05***	0.000***	0.000***	0.000***
*ECS*	0.002***	0.001***	0.002**	0.002***	0.001***	0.002***	0.001***	0.002***	0.002***
*POP*	-0.000**	-0.001*	-0.000**	-0.007	-0.007	-0.002**	-0.000	-0.001***	0.035***
*DIST*	-0.003***	-0.002***	-0.004**	-0.003***	-0.003***	-0.007**	-0.003***	-0.004***	-0.003***
*LANG*	-0.002	-0.007	-0.009	-0.010	-0.008	-0.018**	-0.00255	0.008	-0.000
*PORT*	8.94e-0**	1.12e-05	0.000**	0.000***	0.000***	0.000***	0.003*	5.98e-05**	0.000***
*FDI*	4.86e-05*	5.23e-05	6.47e-05*	8.78e-05*	7.89e-05*	0.000*	2.67e-05	0.001	5.38e-05*
*CONTIG*	0.003	0.169	0.037**	0.030**	0.026**	0.037**	0.00349	-0.024	0.012
*POP ×FDI*		0.000***							
*POP × PORT*			-0.000						
*POP × DIST*.				0.000					
*PORT × FDI*					0.000				
*ECS × DIST*						-0.000			
*ECS × PORT*							-0.000*		
*ECS × FDI*								-0.001***	
*ECS × POP*									-0.002***
AR(2)	0.614	0.676	0.661	0.701	0.680	0.709	0.676	0.553	0.690
Hansen	0.826	0.823	0.865	0.833	0.777	0.720	0.820	0.874	0.837
Prob-F	0.000	0.000	0.000	0.000	0.000	0.000	0.000	0.000	0.000
No. of Group	23	23	23	23	23	23	23	23	23
Observations	195	195	95	195	195	195	195	195	195
Outliers	Excluded	Excluded	Excl195uded	Excluded	Excluded	Excluded	Excluded	Excluded	Excluded
Time effect	Included	Included	Included	Included	Included	Included	Included	Included	Included

Note: ***, **, and * are significant levels at 1, 5, and 10 percent.

**Table 6:** Two-step GMM estimated results of intensive margins of rubber export.

Variables	Model 1 Two-step GMM	Model 2 Two-step GMM	Model 3 Two-step GMM	Model 4 Two-step GMM	Model 5 Two-step GMM	Model 6 Two-step GMM	Model 7 Two-step GMM	Model 8 Two-step GMM	Model 9 Two-step GMM
*L*.*IM*	0.939***	0.890***	0.956***	0.803***	0.741***	0.937***	0.765***	0.905***	0.895***
*SPS*	-1.70e-05	-0.000***	-0.000***	-0.000**	-0.000***	-0.000***	-0.001***	-0.000***	-0.001***
*TBT*	2.41e-05**	0.000**	7.58e-05**	0.000**	0.000*	0.000***	0.000**	0.000***	0.000***
*ECS*	-0.002***	-0.001	-0.00130*	-0.002*	-0.004**	-0.002***	-0.003***	-0.002***	-0.003***
*POP*	0.001***	0.004**	0.003***	0.129***	0.002**	0.001**	0.003***	0.001***	0.158***
*DIST*	0.005***	0.002	0.00321*	0.006**	0.009**	0.007***	0.006***	0.004***	0.006***
*LANG*	0.021***	0.020***	0.00694	0.028***	0.051***	0.017***	0.064***	-1.95e-05	0.0289***
*PORT*	-0.001***	-0.001***	-0.00102**	-0.000***	-0.001***	-0.001***	-0.019***	-0.001***	-0.000*
*FDI*	0.000**	0.000***	0.000283	8.62e-05***	-0.052**	0.000	-0.000	0.010***	7.67e-05
*CONTIG*	-0.021**	-0.034**	-0.026	-0.025		-0.016***	-0.078*	0.0102	-0.020*
*POP × FDI*		-0.002***							
*POP × PORT*			0.001***						
*POP × DIST*.				-0.014***					
*PORT × FDI*					-7.18e-05**				
*ECS × DIST*						-9.58e-05			
*ECS × PORT*							-0.001***		
*ECS × FDI*								-0.001***	
*ECS × POP*									-0.007***
AR(2)	0.371	0.0366	0.372	0.372	0.394	0..371	0.340	0.358	0.364
Hansen	0.382	0.289	0.756	0.666	0.544	0.756	0.420	0.645	0.750
Prob-F	0.000	0.000	0.000	0.000	0.000	0.000	0.000	0.000	0.000
No. of Group	23	23	23	23	23	23	23	23	23
Observations	195	195	195	195	195	195	195	195	195
Outliers	Excluded	Excluded	Excluded	Excluded	Excluded	Excluded	Excluded	Excluded	Excluded
Time Effect	Included	Included	Included	Included	Included	Included	Included	Included	Included

Note: ***, **, and * are significant levels at 1, 5, and 10 percent.

### 4.1. Descriptive statistics of the variables

Table 3 shows the descriptive statistics for the study’s dependent and independent variables. The extensive and intensive margins (*EM* and *IM*) for rubber which are the key dependent variables of interest which have an average value of 0.018, 2.638. The economies of scale (*ECS*) have an average value of 3.13e+08. Other independent variables such as; population size (*POP*), distance (*DIST*), foreign direct investment (*FDI*), technical trade barriers (*TBT*), Sanitary and phytosanitary (*SPS*), each have an average value of 0.711, 8005.2 km, 4.380, 0.413, 0.648 respectively.

In Table 4, the results of the Pearson correlation and variance inflation factor (VIF) are reported to ascertain the degree of multicollinearity among the independent variables. The test indicates that there is no multicollinearity problem. This is because the correlations among the independent variables were low in most cases. Furthermore, the values of the variance inflation factor (VIF) for all the variables are below 5 (i.e. the generally acceptable standard value) and 10 (i.e. the threshold value). For detail see Youssef et al. [45], Abonazel and Shalaby [38], Abonazel and Shalaby [48], Youssef et al. [44], Ibrahim et al. [39], Ibrahim et al. [40], Ibrahim [41], and Ibrahim et al. [42].

After testing for multicollinearity, we also check for outlier problems. This test is important because when there exists an extremely low or high value in the data set which could result in a high residual, the conventional regression estimate of model parameters may produce a biased and unreliable estimator. In our case, we applied Cook’s [49] distance outlier test and identified the outlier observations. These outlier observations were dropped and our valid models were re-estimated. Therefore, based on multicollinearity and outlier test, our dataset only suffers from the problem of outliers which is estimated at 5% (i.e. 12 ÷ 230 × 100 ≈ 5%) see Awwad et al. [50], Dawoud & Abonazel [51]. This problem has been addressed using Cook’s [49] distance outlier test. Cook’s [49] distance outlier test is suitable since we do not have multicollinearity and outlier problems simultaneously which will necessitate the use of the most recent test proposed by Awwad et al. [50], Dawoud & Abonazel [51].

### 4.2. Discussion and results interpretation

This study used panel data which consists the dynamic and spatial panels. The dynamic panel has been estimated using the two-step system GMM method. However, the spatial panel was estimated based on OLS, SAR, and SEM methods on GeoDa. In this study, two dependent variables were utilized in the estimate, which are extensive and intensive margins. For the dynamic panel, based on the Sargan and Arellano-Bond test results, the estimated models are found to be valid and are reported in Tables 5 and 6. Model 1 of Tables 5 and 6 are the baseline models which are estimated without mediation effect.

Findings from Tables 5 and 6 indicate that sanitary and phytosanitary (*SPS*) significantly and negatively affect trade margins. This finding supports the initial hypothesis and is consistent with Jayasinghe et. al. [19]) and Shepotylo’s [15] findings. This negative effect on the rubber trade margins implies that Indonesia is not able to meet the standards imposed by the importing countries or its export destinations countries. Therefore, this causes a decrease in rubber export due to the imposed standard requirements that could result in increased production and transportation cost. The negative effect of the SPS barriers could also result in forcing small and less productive companies out of the export market. For the technical barrier to trade (*TBT*) our estimation strategy revealed a positive and significant effect on both the extensive and intensive margins. This positive nexus exists because Indonesia and local rubber producers were able to meet the requirements of *TBT* imposed by the importing countries. Besides Indonesia is the second leading rubber producer and only second to Thailand. For this reason, and despite the hypothesized negative effect of TBT on trade margins, in the case of Indonesia *TBT* may positively affect trade margins because the required demand by importing countries can only be met with Indonesia’s rubber export. Other empirical studies by Bao and Chen [9], Bao and Qiu [20], and Shepotylo [15] have also supported the positive effect of *TBT* on extensive and intensive margins. The economies of scale (*ECS*) have a significant positive effect on extensive margins and a negative effect on the intensive trade margin. There is no doubt about the positive effect of *ECS* on the extensive margin, this is because higher openness to trade and economies of scale in an export destination country can cause higher demand for imports Kamal and Zaki [52], and Sun and LI [18]. Moreover, large economies of scale owned by the importing country could result in high purchasing power of imported goods leading to more importation.

The population size (*POP*) has a significant negative impact on the extensive margin and a positive impact on the intensive margin. Other being equal, the existence of a negative influence indicates that the high population of partner countries or county of origin (Indonesia) has an impact on Indonesia’s trade margins. This finding is in line with Safriani and Priyarsono [17] and Virginia [21]. The negative correlation is due to overconsumption in the country accompanied by high public purchasing power. Therefore, a slight supply causes a decrease in exports of goods or services abroad. The distance variable (*DIST*) has a significant negative impact on extensive margin and a positive effect on intensive margin. This implies that the distance traveled can increase the production costs of a product so that the price of export goods in the international market will be higher. Moreover, if the distance is relatively low, transportation costs will be lower and hence increase the market share (extensive margins). The language (*LANG*) variable in this study has a negative effect on extensive margins and a positive on intensive margins. Lohmann [53] simply stated that the existence of language barriers between the two countries could reduce the volume of trade. A common language among countries will facilitate trade so that export margins will increase. Besides, different languages can increase trade costs, especially related to communication costs in foreign languages. The *PORT* variable has a significant positive effect on export trade margins and a negative effect on import trade margins. This implies that if there is an increase in the quality of port infrastructure, it will have an increasing impact on export trade margins and decreasing impact on import trade margins. Countries’ trade largely depends on port infrastructure, either land or sea. Improving the quality of infrastructure has a major influence on Indonesia’s trade flows Asikin et al. [54].

Additionally, our empirical finding indicates that *FDI* has a significant positive effect on both trade margins. An increase in the amount of foreign investment can increase the amount of rubber production for export. The incoming investment is used to improve the infrastructure supporting trade activities such as transportation and the construction of rubber product processing factories Farkhan [55]. For the *CONTIG* variable, there exists evidence of a positive and significant effect on extensive margin and a negative effect on intensive margin. In the case of rubber trade margins, the interaction between economies of scale and population size revealed a significant negative effect on both the extensive and intensive margins. While economies of scale with significant distances have a negative and insignificant effect on both margins. The existence of negative effects even though insignificant implies that the distance variable is more dominant in the rubber trade. So, it can be interpreted that the rubber trading activity is somewhat not influenced by the distance between the importing country and the exporter compared to the population size. In the case of the rubber trade margin, the interaction between port quality and *FDI* has a significant negative effect only in the case of intensive margin. The increasing quality of the port, accompanied by an increase in *FDI*, will further strengthen or enhance the growth of the rubber trade margin. An increase in the amount of foreign investment can decrease the intensive margin for Indonesia’s rubber imports. This finding implies that the incoming investment is geared toward substituting rubber imports.

The interaction effects of economies of scale and FDI are negative and significant in both margins. This is because even though the *ECS* value is high and the amount of *FDI* entering the domestic market is large. However, the absence of skills or innovation in processing or utilizing this will have an impact on reducing trade margins. Products produced will be substandard or of low quality. The negative relationship between *FDI* with Indonesia’s export margins is thought to be caused by the concept of a product’s life cycle Vernon [56].

Based on the estimated results from the GMM, findings show that for both the extensive and intensive margin the p-value corresponding to F-statistic is 0.000 which is highly significant at less than 1 percent level. This indicates a joint significance of the explanatory variables in influencing the dependent variable.

### 4.3. Spatial estimation

In this part of the analysis, the study also analyzed the spatial aspects of the independent variables on the dependent variables. In spatial aspects, we considered the existence of space between countries. The existence of spatial aspects between countries or variables indicates that there is an intense connection between the countries. In this study, the variables used to detect the spatial aspects are the *ECS*, *POP*, *PORT*, and *FDI*. Spatial aspects in this study are based on weighting using GeoDa, and include queen contiguity, as a weighting in the estimation process.

**Fig 1:**
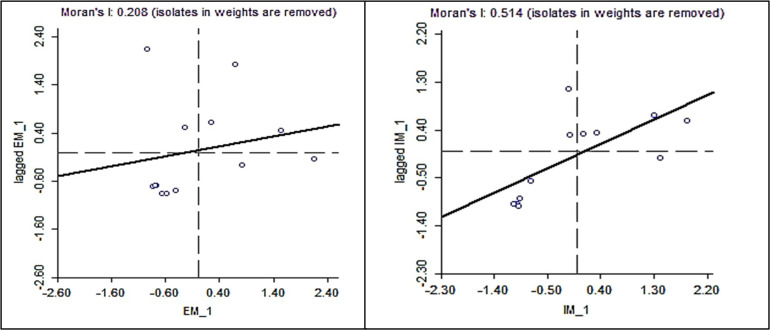
Spatial trade margin interactions using Moran I test (Moran Scatterplot).

In Moran I, the test is used to see the spatial effect as depicted in Fig 1. Moran, I show a positive value of 0.208 for extensive margins and 0.514 for the intensive margin. The greater the value of Moran, the higher the spatial relationship between adjacent states. Positive values indicate that regions with high spatial *EM* and *IM* values are surrounded by regions with high values as well. To detect the presence or absence of spatial aspects a static or spatial panel regression analysis is certainly needed to support or strengthen the spatial aspects. Spatial regression methods can be done by selecting the best model between the classical model (OLS), spatial autocorrelation model (SAR), or spatial error model (SEM).

In Table 7, the selected model is the SEM model. The SEM model has an R Square value greater than the other models and the smallest AIC value. The R^2^ for the SEM is 0.84, while SAR has an R^2^ of 0.31. Therefore, SEM is the model with the highest R^2^ and the best model. In the SEM model, only the *ECS* and *POP* variables have a significant effect on the intensive margin. This shows that the *ECS* and *POP* variables as a proxy for demand factors are more dominant from the spatial aspects of trade margins. This finding is consistent with Hamzalouh et al. [57], Porojan [58], and Quang et al. [59] who show that economies of scale and population size have a spatial influence on trade margins. Based on the analyses of the spatial panel data in Table 7, it can be seen that the p-value of the spatial dependency test is 0.000 < 0.05 so it rejects H_0_. This means that there are spatial dependencies in our cross-sectional data. Thus, the agglomeration of the country covered in this study shows that the rubber commodity trade is more dominant in countries with close distances.

**Table 7:** Results of spatial model estimates.

	**Dependent variable (IM)**			**Dependent variable (EM)**		
	OLS	SAR	SEM	OLS	SAR	SEM
*lnECS*	1.874	1.874	18.572***	-0.003	-0.011	-0.013
*POP*	1.840	1.840	8.660*	-0.013	-0.011	0.004
*FDI*	0.095	0.0950	0.113	0.000	9.02763e-1	0.000
*PORT*	-9.971	-9.971	-3.539	-0.010	-0.005	-0.004
**Diagnostics test**						
R^2^	0.17	0.31	0.84	0.33	0.71	0.81
AIC	150.9	152.9	136.1	-96.4	-98.4	-100.9
*Likelihood ratio test*	1.568	0.000	4.462***	0.899	3.967	14.793***
The recommended model is SEM						

Note: ***, **, and * are significant levels at 1, 5, and 10 percent

## 5. Conclusion and policy implication

This study scrutinizes the determinant of extensive and intensive margins of rubber product exporters in Indonesia over the period 2009–2018. A sample of 23 countries that mostly import Indonesia’s rubber products was analyzed using dynamic short panel data with a two-step system generalized method of moment (GMM). A special emphasis has been paid to the role of non-tariff measures such as sanitary and phytosanitary (SPS), technical barriers to trade (TBT), and gravity factors in determining rubber trade margins. Our empirical strategies uncover that sanitary and phytosanitary policies negatively affect trade margins. This causes exporters to reduce rubber export and further result in a low level of rubber productivity. Due to this adverse effect, smaller exporters in the rubber industry can leave the market because of high standards or obstacles from importing countries. Technical trade barriers were found to have increased trade margins. This implies Indonesia’s rubber exporters can meet the standards imposed by importing countries. As regards the foreign direct investment (FDI) our finding established strong evidence that more FDI is largely associated with increased trade margins. However, economies of scale, port, and contiguity increase extensive margin and reduce intensive, population size, distance, and language barrier reduce extensive margin and increase intensive margin. The influence of population size which saw an increase in intensive margin could be due to overconsumption in the country, accompanied by high purchasing power. Further evidence revealed that high population size and port quality accompanied by high FDI and distance increases extensive margin and reduces intensive margin. High economics of scale accompanied by distance, port quality, FDI, and population size reduces both trade margins. Our empirical strategy from the spatial analysis does not give overall significant results on each variable as only economies of scale and population size seem to have a spatial influence on trade margins. In this vein, the finding indicates that from the side of exports and in terms of the rubber trade margin, there are spatial effects. This also implies the existence of spatial aspects and agglomeration in areas that are close together.

From the findings, SPS largely and negatively affects trade margins. We, therefore, recommend that innovation both in terms of technology, like industrial innovation in the field of rubber processing and certification related to rubber commodities, needs to be increased to intensify and expand Indonesia’s rubber market share. Since the findings from the spatial aspects have not yielded overall significant results for all the variables, we recommend that future studies should incorporate other commodities and other variables to support the hypothesis based on the significance of the results of the spatial panel. There is an additional need to improve the quantity and quality of rubber export commodities to increase or widen rubber exports to other destination countries. To increase the demand for rubber there is the need to provide training related to quality standards applied by destination countries to increase exports. Besides that, training or technological innovation in processing rubber products’ quality or skills of workers can increase along with the increasing population. There is a need to improve infrastructure and modes of transportation. This is needed to reduce trade costs. In addition, quality improvement or fulfillment of destination country standards needs to be improved so that trade barriers, both tariffs and non-tariffs, can be overcome.

### 5.1 Limitations of the study

The major weakness of this study lies in its focus on the analysis of the impact of non-tariff barriers to trade on the export and import of rubber trade flows as measured by the extensive and intensive margins over the period 2009–2018. Another limitation of this study centered on the limited sample covering only 23 export and import destinations. The study was also based on short panel data analysis. With this, therefore, future work should improve on this study by first focusing on both tariff and non-tariff measures’ impact on Indonesia’s trade margins of not only rubber products but many different commodities. Secondly, there is also the need to cover not only major export and import destinations but rather many destination countries that are strategic to Indonesia’s trade and foreign policy. In addition to this, future studies should also bring to light findings from the use of long panel data analysis concerning the trade margins of Indonesia’s rubber export.

## Supporting information

S1 Data (XLSX)
